# The New Antipyretic

**Published:** 1894-03

**Authors:** 


### Selections.

### THE NEW ANTIPYRETIC.

### RECENT APPLICATIONS OF GUAIACOL.

Some recent clinical applications of guaiacol invite a consideration of its chemic and therapeutic relations. "Guaiacol is a substance that occurs in wood-tar, and is also produced by heating pyrocatechin and potassium methyl-sulphate to 180 degrees, C. It is a colorless liquid that boils at 200 degrees C., and has a specific gravity of 1117. It is readily soluble in alcohol, ether and acetic acid, and is recommended instead of creasote in pulmonary tuberculosis."* This substance is a prominent constituent of the best creasote, into which it enters to the extent of about 60 per cent., although in the cheaper kinds it may be much less than this. Along with the guaiacol, are associated a number of more or less objectionable substances. Among these, according to Allen, are phenol, paracresol, cresol, kylenol, creasol, homocreasol, cœrneignol, and four methylic ethers of trihydric phenols. Many of the foregoing are distinctly toxic, but pure creasote, Huseman declares to be non-poisonous. Those who are in a position to know about it, however, state positively that

* Definition from advance sheets of Gould's "New Medical Dictionary."

there is much poisonous creasote in the market.† The "United States Dispensatory" states that: "Commercial creasote almost always contains carbolic and cresylic acids from coal tar; and indeed much of what is sold for creasote is nothing more than impure carbolic acid." This is not written in order to discredit the use of pure beeech-wood creasote in medicine, as originally suggested by Sommerbrodt; but, to explain why chemists and clinicians have diligently sought for a similar preparation possessing equally beneficial effects, but having definite and uniform composition. Thus creasotal, or creasote carbonate was brought out by Dr. Chaumier in a paper read before the Academy of Medicine of Paris.‡ Dr. F. Walzer reported favorably upon benzosol, (a compound of benzoic acid with pure guaiacol, forming benzoylorrho-öxyanisol) which has the advantage over the preceding in the fact that it is a solid, being a colorless crystalline powder almost free from smell and taste. In the digestive tract it splits up again into its constituents, guaiacol and benzoic acid.

It might also be of interest to know that recently Drs. A. Gilbert and L. Maurat have reported favorably upon the effects of a chemically pure form of guaiacol, which is made artificially, (synthetic guaiacol). This substance appears in the form of white, rhombohedial crystals fusing at 28.5 degrees C., and boiling at 20.5 degrees C., with a specific gravity of 1.143. It is practically insoluble in water, but is soluble in alcohol, oils, and anhydrous glycerin. It possesses a sweet taste at first, which soon changes to a pricking and burning sensation. § It melts into a sticky paste at the temperature of the surface of the body. This has been used, combined with oil, in capsules, for internal administration in phthisis principally.

Drs. Weil and Diamantberger, since 1889 have administered pure guaiacol in sweet almond oil (equal parts of each) by subcutaneous injection, in all the forms of pulmonary tuberculosis, and claim to have had very good results. || Of eighty-two patients, sixty-two were improved, two remained stationary, and eighteen became worse while under this treatment. Of the sixty-two cases, twenty-seven were considered cured. In the

---

† Nagelvoort on "Poisonous Creasote," Bulletin of Pharmacy, Vol. vi, page 557.

‡ Deutsche Med. Wochenscrift, 1893, Nos. 24–25.

§ The Medical Week.

|| Bulletin Medical, 1893. No. 68.

majority of cases the unfavorable symptoms of the disease were strikingly modified after a relatively short time.

About a year ago, Dr. Sciolia * made the remarkable observation that external applications of guaiacol to a small area of the general surface, were promptly and uniformly followed by a reduction of the bodily temperature. This was soon corroborated by the results obtained in tuberculosis cases by Bard, and also by Lannois. Subsequently Robillard of Lille † also repeated these observations upon guaiacol, and confirmed Sciolla's statements concerning its powerful antipyretic effect. Drs. Cassovice and Sigalea modified Sciolla's method by not applying the remedy in a pure state, but in combination with the tincture of iodine (one part guaiacol to five parts of iodine). Of this, about 2 drachms are used for a single painting, applied on the affected side all along the posterior part of the throat, every evening. These paintings are said to give rise to a considerable fall in temperature, abundant perspiration, and increased diuresis. In a severe case of left pleuritic effusion, in which puncture did not bring about any improvement, the fever disappeared, and after a few applicatiions, the extravasation was promptty absorbed.‡

There are many surprises among the untoward effects of drugs, and what these may be in the case of guaiacol remains to be seen. In the meantime, however, therapeutics has been enriched by a potent and (as the rule at least), non-poisonous antipyretic possessing decided advantages over similar substances obtained from among the aromatic series of coal-tar products.—*Journal American Medical Association.*

---

### A BILL TO ESTABLISH A DEPARTMENT OF PUBLIC HEALTH.

*Be it enacted by the Senate and House of Representatives of the United States in Congress assembled:*

SECTION 1. That there shall be established a Department of Public Health. There shall be appointed by the President from the medical profession, by and with the advice and consent of

---

* La Semaine Medicale, 1893. No. 21.

† Societe de Biologie, July, 1893.

‡ American Med. Surg. Bulletin, November, 1893; from Semaine Medicale No. 52.

